# Modeling the “F” in “SAFE”: The dynamic game of facial cleanliness in trachoma prevention

**DOI:** 10.1371/journal.pone.0287464

**Published:** 2023-06-23

**Authors:** Mary Barazanji, Janesah D. Ngo, Jule A. Powe, Kimberley P. Schneider, Jan Rychtář, Dewey Taylor

**Affiliations:** 1 Department of Kinesiology and Health Sciences, Virginia Commonwealth University, Richmond, VA, United States of America; 2 Department of Biology, Virginia Commonwealth University, Richmond, VA, United States of America; 3 Department of Mathematics and Applied Mathematics, Virginia Commonwealth University, Richmond, VA, United States of America; 4 Department of Chemistry, Virginia Commonwealth University, Richmond, VA, United States of America; University of California San Francisco School of Medicine, UNITED STATES

## Abstract

Trachoma, a neglected tropical disease (NTDs) caused by bacterium *Chlamydia trachomatis*, is a leading cause of infectious blindness. Efforts are underway to eliminate trachoma as a public health problem by using the “SAFE” strategy. While mathematical models are now standard tools used to support elimination efforts and there are a variety of models studying different aspects of trachoma transmission dynamics, the “F” component of the strategy corresponding to facial cleanliness has received very little attention so far. In this paper, we incorporate human behavior into a standard epidemiological model and develop a dynamical game during which individuals practice facial cleanliness based on their epidemiological status and perceived benefits and costs. We found that the number of infectious individuals generally increases with the difficulty to access a water source. However, this increase happens only during three transition periods and the prevalence stays constant otherwise. Consequently, improving access to water can help eliminate trachoma, but the improvement needs to be significant enough to cross at least one of the three transition thresholds; otherwise the improved access will have no noticeable effect.

## Introduction

Trachoma is a neglected tropical disease (NTDs) caused by bacterium *Chlamydia trachomatis* [1]. Repeated infection eventually leads to conjunctival scarring, and blindness [2], making trachoma the leading cause of infectious blindness [1].

From 1993, the World Health Organization (WHO) recommended using the “SAFE” strategy to eliminate trachoma as a public health problem [1]. The strategy relies on Surgery to treat the blinding stage, Antibiotics and particularly the mass drug administration of the antibiotic azithromycin, Facial cleanliness, and Environmental changes, particularly improving access to water and sanitation such as increasing access to well-designed latrines that helps to reduce the population density of flies [3].

Through the Alliance for the Global Elimination of Trachoma by 2020 (GET2020), WHO was aiming to eliminate trachoma as a public health problem by 2020. One goal of this initiative was to to reduce the prevalence of trachomatous inflammation, follicular (TF) in children aged 1–9 years, to less than 5% by 2020 [4, 5]. The NTDs road map 2021–2030 sets 2030 as the new target date [6].

The risk of active trachoma is associated with the presence of the eye-seeking flies *Musca sorbens* on the face [7]. The flies are a known vector for trachoma in sub-Saharan Africa [8]; although the transmission can also occur by a direct or indirect contact with an infected person [2]. The females seek out protein-rich human ocular discharge and as they move from eye to eye, they transmit *Chlamydia trachomatis* [9, 10]. The bacteria can live for up to 6 hours in the flies’ intestines and for up to 2 hours on their legs [11]. On the other hand, the flies may not be transmitting trachoma in other regions such as Brazil where trachoma is also endemic [12].

Recently, there have been efforts to scale-up the facial cleanliness and environmental improvement (F&E) components of the SAFE strategy [13]. As noted in [13], improving understanding of the F&E interventions could lead to more effective and sustained changes.

Mathematical modeling is now a standard tool for NTDs elimination efforts [14] and there are many different models of trachoma [15]. [16] reviews 23 articles on mechanistic or statistical modeling of the transmission, dynamics and/or control of trachoma. Most of the models adopt Susceptible-Infected-Susceptible (SIS) structure [17–23] and focus on the Polymerase Chain Reaction (PCR) detectable infectious stage [24]. However, individuals can remain TF positive with detectable disease for far longer than they are PCR positive [25]. Models in [24–26] all captured the dynamics of both the PCR and TF positivity that is the most relevant to the WHO elimination goal. The impact of COVID-19 on control and elimination of trachoma was considered in [5] and the prediction of prevalence based on various data was modeled in [27].

Most of the previous models focus on the MDA; and very little modeling has been done to consider the effect of nonpharmaceutical interventions (and particularly the F and E components of the SAFE strategy) [16].

Moreover, current models of trachoma ignore human behavior completely. [28–30] and later [31, 32] introduced vaccination games and incorporated voluntary disease prevention into standard epidemics modeling to provide more insight and better predictions [33]. Thus, game theory components are now increasingly added to epidemiological models [34] and can serve as predictive tools in populations for extracting an optimum decision-making strategy [35].

In this paper, we incorporate human behavior into an epidemiological model of trachoma. We adopt a model developed by [24] and allow individuals practice facial cleanliness based on their epidemiological status and their perceived benefits and costs. We investigate how the optimal behavior depends on various parameters, most notably the cost of water and the number of flies per person.

## Model

We will incorporate the facial cleanliness component into a model from [24] who investigated four different age structured models of trachoma transmission. Their model number 2 included the standard SEIRS (Susceptible-Exposed-Infectious-Recovered-Susceptible) structure and provided the best fit to trachoma infections data. We will extend their model by assuming that a fraction of individuals in each compartment practice cleanliness.

**Fig 1:**
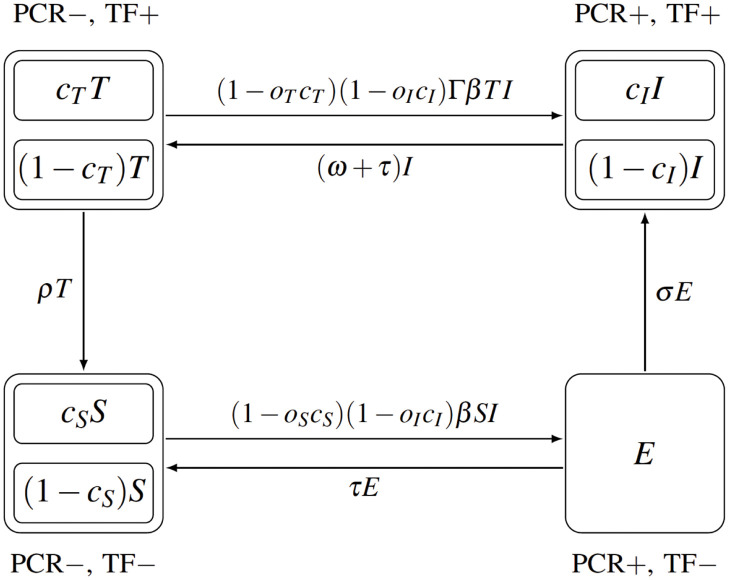
Transmission of trachoma. The population is divided into susceptible (*S*), exposed (*E*), infectious (*I*), and individuals with follicular trachomatous inflammation (*T*). The compartments *S*, *I* and *T* are further divided into individuals that practice cleanliness and those who do not (the same division of *E* is possible but irrelevant). The location of the compartments in the diagram indicates the test status: on the left is PCR negative, on the right PCR positive, top TF positive, bottom TF negative. The arrows represent transitions between the compartments. The parameter notation is explained in Table 1. This model extends model 2 presented in [24] with the graphical arrangement as in [25].

We consider a closed population with no births, deaths, or migration. The population is divided into four groups based on the results of PCR and TF tests; see also Fig 1.

1. Susceptible (*S*) are PCR negative and TF negative,
2. Exposed (*E*) are PCR positive and TF negative; we assume the exposed individuals do not spread the disease,
3. Infectious (*I*) are PCR positive and TF positive; they can spread the disease to other people either directly or through the flies,
4. Cases with trachomatous inflammation, follicular (TF) (*T*); they are PCR negative and TF positive. They cannot spread the disease, usually returning to being susceptible, but they can become reinfected and go directly back to *I*.

Since we consider a closed population, the letters *S*, *E*, *I*, *T* will represent the proportion of the whole population in a particular group.

All individuals can practice cleanliness and the level of cleanliness will have an effect on the transmission dynamics. However, we will first describe the dynamics as if nobody practices cleanliness and add the cleanliness component later.

### Underlying compartmental model without cleanliness

Susceptible individuals, *S*, test PCR negative and TF negative. They can be exposed through two primary routes [16]: (1) a direct contact with an infected individual or with a clothing that touched discharge from an infected person’s eyes or nose [2], or (2) through eye-seeking flies (e.g., *Musca sorbens*) which have contacted the infectious discharge [9]. Thus, we assume that the force of infection, i.e., the rate at which they individuals become exposed, is given by

$$\begin{array}{c} \lambda =(\beta_{H}+\beta_{F}\left(n_{F}\right))I \end{array}$$ (1)

where *β*_*H*_ is the transmission rate via contact with another individual and/or their clothing, *β*_*F*_(*n*_*F*_) is the transmission rate through the flies when there are *n*_*F*_ flies per person. We assume

$$\begin{array}{c} \beta_{F}\left(n_{F}\right)=\beta_{F}^{\text{max}}\frac{n_{F}}{n_{F}+n_{F,1/2}}, \end{array}$$ (2)

where $\beta_{F}^{\text{max}}$ is the maximum transmission rate from flies, and *n*_*F*,1/2_ is the number of flies needed to achieve half of the maximal transmission rate; $\frac{n_{F}}{n_{F}+n_{F,1/2}}$ is the Holling type II function [36]. We will denote

$$\begin{array}{c} \beta =(\beta_{H}+\beta_{F}^{\text{max}}\frac{n_{F}}{n_{F}+n_{F,1/2}}) \end{array}$$ (3)

and thus have λ = *βI*.

The exposed individuals, *E*, test PCR positive and TF negative. They are not infectious but incubate the disease for a period *σ*^−1^.

After the incubation period, the individuals become infectious (*I*) and test PCR positive and TF positive. The infectious stage lasts for a time *ω*^−1^ and then the infectious individuals develop follicular trachomatous inflammation (*T*).

Individuals in *T* are no longer infectious and test PCR negative yet they are TF positive. They also have partial immunity for reinfection. After a contact with an infectious individual, their clothing or through a fly, they can become reinfected and return to *I* at the reduced rate Γλ where Γ ∈ [0, 1] is the susceptibility to reinfection.

The follicular inflammation lasts for the time *ρ*^−1^ and after that the individuals become susceptible again.

Finally, we assume that the whole population is treated by antibiotics at rate *τ*. The antibiotic treatment changes the PCR status to negative but leaves the TF status unchanged. Thus, as in [24], after the treatment, individuals move from *E* to *S* and from *I* to *T*.

### Underlying compartmental model with facial cleanliness

Here we assume that individuals practice facial cleanliness and describe the effect of it on the transmission dynamics.

The cleanliness of infectious individuals will reduce their infectiousness. Similarly, the cleanliness of susceptible individuals will reduce their chances of getting exposed and the cleanliness of cases with follicular inflammation will reduce their chances for reinfection. At the same time, we will assume that cleanliness has no or limited effect on other parts of the dynamics. Specifically, we will assume that the cleanliness status does not influence incubation period. Similarly, we assume the cleanliness of individuals in *T* does not influence the duration of the follicular inflammation. Moreover, since individuals in *E* already are PCR positive and yet cannot spread the disease, their cleanliness status does not influence any part of the dynamics.

Mathematically, to incorporate the cleanliness into the model, we use *c*_*X*_ ∈ [0, 1] to denote the proportion of individuals in a compartment *X* ∈ {*S*, *I*, *T*} that practice cleanliness; based on the above assumptions, we do not need to consider *c*_*E*_.

Furthermore, let *o*_*I*_ denote the reduction of infectivity due to the cleanliness; i.e., individuals in *I* that practice cleanliness spread the disease (1 − *o*_*I*_) times less than the individuals that do not practice cleanliness. Similarly, let *o*_*S*_ and *o*_*T*_ denote the reduction of susceptibility to exposure and reinfection, respectively, i.e., individuals in *S* that practice cleanliness get exposed (1 − *o*_*S*_) times less likely than individuals who do not practice cleanliness and individuals in *T* get reinfected (1 − *o*_*T*_) less likely if they practice cleanliness versus if they don’t.

We define

$$\begin{array}{cc} r_{I} & =1-o_{I}c_{I} \end{array}$$ (4)

$$\begin{array}{cc} r_{S} & =1-o_{S}c_{S} \end{array}$$ (5)

$$\begin{array}{cc} r_{T} & =1-o_{T}c_{T} \end{array}$$ (6)

The term *r*_*I*_ = (1 − *c*_*I*_) + (1 − *o*_*I*_)*c*_*I*_ signifies that the fraction 1 − *c*_*I*_ of infectious individuals is “fully infectious” while the fraction *c*_*I*_ of individuals that practice cleanliness has the infectiousness reduced by a factor *o*_*I*_ to (1 − *o*_*I*_). Consequently, the force of infection is reduced from *βI* to λ = *r*_*I*_*βI*.

Similarly, the term *r*_*S*_ = (1 − *c*_*S*_) + (1 − *o*_*S*_)*c*_*S*_ signifies that the fraction (1 − *c*_*S*_) of susceptible individuals are fully susceptible while a fraction *c*_*S*_ of *S* has the susceptibility reduced by a factor (1 − *o*_*S*_). The rate at which the susceptible individuals get exposed is thus *r*_*S*_*r*_*I*_*βSI*. The meaning of the term *r*_*T*_ is analogous.

The model yields the following system of differential equations.

$$\begin{array}{c} \frac{dS}{dt}=\rho T+\tau E-\left(1-o_{S}c_{S}\right)\left(1-o_{I}c_{I}\right)\beta SI \end{array}$$ (7)

$$\begin{array}{c} \frac{dE}{dt}=\left(1-o_{S}c_{S}\right)\left(1-o_{I}c_{I}\right)\beta SI-\left(\sigma +\tau \right)E \end{array}$$ (8)

$$\begin{array}{c} \frac{dI}{dt}=\sigma E+\left(1-o_{T}c_{T}\right)\left(1-o_{I}c_{I}\right)\Gamma \beta TI-\left(\omega +\tau \right)I \end{array}$$ (9)

$$\begin{array}{c} \frac{dT}{dt}=\left(\omega +\tau \right)I-[\left(1-o_{T}c_{T}\right)\left(1-o_{I}c_{I}\right)\Gamma \beta I+\rho ]T \end{array}$$ (10)

The notation is summarized in Table 1 and the model diagram is shown in Fig 1.

**Table 1:** Model parameters. The times are in weeks, rates are per capita per week. The explanation of how the values were derived is provided in the Calibration Section.

Notation	Meaning	Base value	Range	Reference(s)
*σ* ^−1^	Incubation period	2.5	[1.5, 4]	[25]
*ρ* ^−1^	Duration of the post-infection inflammatory disease	5.4	[3.6, 8]	[25]
*ω* ^−1^	Duration of the infectious period	15	[11, 20.5]	[25]
*β*	Cumulative transmission rate	(3)	-	Assumed
*β*_*H*_	Direct transmission rate through infectious individual	0.43	[0.35, 0.58]	[24]
$\beta_{F}^{\text{max}}$	Indirect transmission rate through flies	*β*_*H*_	-	Assumed
*n*_*F*_	Number of flies per person	2	[0, 5]	Assumed
*n*_*F*,1/2_	Number of flies per person needed to achieve 50% of the indirect transmission rate	2	[0, 5]	Assumed
Γ	Reduction in susceptibility in *T*	0.5	[0.2, 0.8]	[24]
*o*_*S*_	Reduction in susceptibility when practicing cleanliness in *S*	0.76	[0.57, 0.9]	[37]
*o*_*T*_	Reduction in susceptibility to reinfection when practicing cleanliness in *T*	*o*_*S*_	-	Assumed
*o*_*I*_	Reduction of infectivity when practicing cleanliness in *I*	*o*_*S*_	-	Assumed
*τ*	Rate of MDA	0	-	Assumed
*C*_*W*_	Cost of water for cleaning	0.5	[0, 10]	Assumed
*κ*_*S*_	Proportionality constant to convert exposure rate into the cost for an individual in *S*	20	-	Assumed
*κ*_*T*_	Proportionality constant to convert reinfection rate into the cost for an individual in *T*	*κ*_*S*_	-	Assumed
*κ*_*I*_	Proportionality constant to convert rate of infecting others into the cost for an individual in *I*	0.01	-	Assumed
*κ*′	Scaling factor for the behavioral dynamics	10	-	Assumed
*ϕ*	Conversion factor to evaluate discomfort caused by flies as *ϕn*_*F*_	0.1	[0, 0.15]	Assumed

### Game-theoretical component

Above, we considered the parameters *c*_*S*_, *c*_*T*_ and *c*_*I*_ to be fixed constants. Here we add the game-theoretic components by assuming that individuals can decide whether or not to practice cleanliness and thus, in turn, influence the values of these parameters. We incorporate such a choice into the model in a similar fashion as done in [32], see also for example [38–42].

As explained in [32], individuals (or their parents) play the game not only against individuals in the current generation, but also against previous generations of behaviorally identical individuals (and their parents). This is a consequence of the fact that the current prevalence of trachoma in the population is partly determined by the history of behavior in the population. Hence, the payoff of individuals not practicing the cleanliness depends implicitly upon the strategy history and relevant behavioral and epidemiological parameters.

In each compartment *X* ∈ {*S*, *I*, *T*}, the individuals have to choose one of the two strategies, to practice cleanliness or not. There is a potential cost and benefit to either choice. In regions where trachoma is endemic, water is a relatively scarce resource. Thus, practicing cleanliness comes at a cost *C*_*W*_ (per unit of time) which could be understood in terms of difficulty getting the water (and the inability to use it for other needs after washing). And since cleanliness does not protect fully against trachoma, there is still the risk of contracting it which depends on the state of the individual. Similarly, the cost of not practicing cleanliness depends on the stage of the individual. In *S* and *T*, the cost is a risk of contracting trachoma. In *I*, the cost is the risk of infecting others and also the discomfort caused by the flies.

In *S*, the susceptible individuals become exposed either at the rate (1 − *o*_*I*_*c*_*I*_)*βI* if they do not practice cleanliness, or at the rate (1 − *o*_*S*_)(1 − *o*_*I*_*c*_*I*_)*βI* if they do. Following [32], we assume the cost is proportional to that rate. Putting it all together, if an individual in *S* practices cleanliness, their cost is

$$\begin{array}{cc} C_{c,S}=C_{W}+\kappa_{S}\left(1-o_{S}\right)\left(1-o_{I}c_{I}\right)\beta I, & \text{if}\;\text{they}\;\text{practice}\;\text{cleanliness,} \end{array}$$ (11)

and

$$\begin{array}{cc} C_{d,S}=\kappa_{S}\left(1-o_{I}c_{I}\right)\beta I, & \text{if}\;\text{they}\;\text{don}’\text{t}\;\text{practice}\;\text{cleanliness.} \end{array}$$ (12)

The costs for the individuals in *T* are analogous, only the rate of reinfection is reduced by a factor Γ. Thus, the costs are

$$\begin{array}{cc} C_{c,T}=C_{W}+\kappa_{T}\left(1-o_{T}\right)\left(1-o_{I}c_{I}\right)\Gamma \beta I, & \text{if}\;\text{they}\;\text{practice}\;\text{cleanliness,} \end{array}$$ (13)

and

$$\begin{array}{cc} C_{d,T}=\kappa_{T}\left(1-o_{I}c_{I}\right)\Gamma \beta I, & \text{if}\;\text{they}\;\text{don}’\text{t}\;\text{practice}\;\text{cleanliness.} \end{array}$$ (14)

Finally, the individuals in *I* are already infected and thus do not have to worry about prevention. However, flies are attracted to the discharge [9] and consequently the cost of not practicing cleanliness is proportional to the number of flies. Also, in the presence of an educational campaign explaining transmission of trachoma, the individual may worry about infecting others, especially in their own family, related to the inclusive fitness [43]. We assume the inclusive fitness part of the cost is proportional to *S* + Γ*T*, i.e., the proportion of population that can still be infected. Hence, the costs are

$$\begin{array}{cc} C_{c,I}=C_{W}+\kappa_{I}\left(1-o_{I}\right)\beta \left(S+\Gamma T\right), & \text{if}\;\text{they}\;\text{practice}\;\text{cleanliness,} \end{array}$$ (15)

and

$$\begin{array}{cc} C_{d,I}=\kappa_{I}\beta \left(S+\Gamma T\right)+\phi n_{F}\; & \text{if}\;\text{they}\;\text{don}’\text{t}\;\text{practice}\;\text{cleanliness.} \end{array}$$ (16)

The imitation dynamics is then constructed as follows. We assume that individuals randomly sample other members of the population at some constant rate. If the strategy of the sampled member provides a higher payoff, then their strategy is adopted with a probability proportional to the expected gain in payoff. For individuals in *S*, the expected gain of switching from not practicing to practicing cleanliness is Δ*C*_*S*_ = *C*_*d*,*S*_ − *C*_*c*,*S*_. Similarly, the expected gain in *T* is Δ*C*_*T*_ = *C*_*d*,*T*_ − *C*_*c*,*T*_ and the individuals in *I*, the expected gain is Δ_*I*_ = *C*_*d*,*I*_ − *C*_*c*,*I*_. This yields the following differential equations for *c*_*S*_, *c*_*T*_, *c*_*I*_.

$$\begin{array}{c} \frac{dc_{S}}{dt}=\kappa^{\prime }c_{S}\left(1-c_{S}\right)(\kappa_{S}o_{S}\left(1-o_{I}c_{I}\right)\beta I-C_{W}) \end{array}$$ (17)

$$\begin{array}{c} \frac{dc_{T}}{dt}=\kappa^{\prime }c_{T}\left(1-c_{T}\right)(\kappa_{T}o_{T}\Gamma \left(1-o_{I}c_{I}\right)\beta I-C_{W}) \end{array}$$ (18)

$$\begin{array}{c} \frac{dc_{I}}{dt}=\kappa^{\prime }c_{I}\left(1-c_{I}\right)(\kappa_{I}o_{I}\beta \left(S+\Gamma T\right)+\phi n_{F}-C_{W}) \end{array}$$ (19)

where *κ*′ > 0 is a time scaling factor that makes the adjustments of *c*_*S*_, *c*_*T*_, and *c*_*I*_ happen on a much faster scale than the trachoma dynamics (7)–(10).

## Analysis of the ODE model

### Disease-free equilibria

Let us focus on the disease-free equilibrium first, i.e. the situation when (*S*, *E*, *I*, *T*) = (1, 0, 0, 0). Any of the eight combinations *c*_*S*_ ∈ {0, 1}, *c*_*T*_ ∈ {0, 1}, and *c*_*I*_ ∈ {0, 1} is an equilibrium of the system (17)–(19).

However, in the DFE, $\frac{dc_{S}}{dt}<0$ and $\frac{dc_{T}}{dt}<0$. Hence, *c*_*S*_ = 0 and *c*_*T*_ = 0 are stable, while *c*_*S*_ = 1 and *c*_*T*_ = 1 are unstable.

Which value of *c*_*I*_ ∈ {0, 1} is stable depends on the sign of Δ*C*_*I*_ = *κ*_*I*_*o*_*I*_*β*(*S* + Γ*T*) + *ϕn*_*F*_ − *C*_*W*_. If Δ*C*_*I*_ > 0, in particular when

$$\begin{array}{c} \kappa_{I}o_{I}\beta +\phi n_{F}-C_{W}>0 \end{array}$$ (20)

and especially if

$$\begin{array}{c} \phi n_{F}>C_{W} \end{array}$$ (21)

then *c*_*I*_ = 1 is stable. On the other hand, *c*_*I*_ = 0 is stable when *κ*_*I*_*o*_*I*_*β* + *ϕn*_*F*_ − *C*_*W*_ < 0.

### Reproduction number and stability of DFE

To determine the stability of the disease-free equilibria, we will calculate the effective reproduction number using the next-generation matrix method [44].

There are three compartments carrying the infection, *E*, *I*, *T* and we will keep them in this order. The rate of new infections is given by

$$\begin{array}{c} F=[\begin{array}{c} \left(1-o_{S}c_{S}\right)\left(1-o_{I}c_{I}\right)\beta I\\ 0\\ 0 \end{array}]. \end{array}$$ (22)

By differentiating F at the disease-free equilibrium we obtain

$$\begin{array}{c} F=[\begin{array}{ccc} 0 & \left(1-o_{S}c_{S}\right)\left(1-o_{I}c_{I}\right)\beta & 0\\ 0 & 0 & 0\\ 0 & 0 & 0 \end{array}]. \end{array}$$ (23)

The other transmissions in the system are given by

$$\begin{array}{c} V=[\begin{array}{c} -(\sigma +\tau )E\\ \sigma E+\left(1-o_{T}c_{T}\right)\left(1-o_{I}c_{I}\right)\Gamma \beta TI-\left(\omega +\tau \right)I\\ \left(\omega +\tau \right)I-(\left(1-o_{T}c_{T}\right)\left(1-o_{I}c_{I}\right)\Gamma \beta I+\rho )T \end{array}]. \end{array}$$ (24)

By differentiating V at the disease-free equilibrium we obtain

$$\begin{array}{c} V=[\begin{array}{ccc} -(\sigma +\tau ) & 0 & 0\\ \sigma & -(\omega +\tau ) & 0\\ 0 & \omega +\tau & -\rho \end{array}]. \end{array}$$ (25)

Thus,

$$\begin{array}{c} V^{-1}=[\begin{array}{ccc} -\frac{1}{\sigma +\tau } & 0 & 0\\ -\frac{\sigma }{\left(\sigma +\tau )(\omega +\tau \right)} & -\frac{1}{\omega +\tau } & 0\\ -\frac{\sigma }{(\sigma +\tau )\rho } & -\frac{1}{\rho } & -\frac{1}{\rho } \end{array}] \end{array}$$ (26)

and

$$\begin{array}{c} FV^{-1}=\left(1-o_{S}c_{S}\right)\left(1-o_{I}c_{I}\right)\beta [\begin{array}{ccc} -\frac{\sigma }{\left(\sigma +\tau )(\omega +\tau \right)} & -\frac{1}{\omega +\tau } & 0\\ 0 & 0 & 0\\ 0 & 0 & 0 \end{array}]. \end{array}$$ (27)

The largest eigenvalue of *FV*^−1^ is

$$\begin{array}{c} R=\rho (FV^{-1})=\left(1-o_{S}c_{S}\right)\left(1-o_{I}c_{I}\right)\beta (\frac{\sigma }{\sigma +\tau })(\frac{1}{\omega +\tau }). \end{array}$$ (28)

The formula (28) could be derived directly from the Fig 1 considering that an individual stays in *I* for time (*ω* + *τ*)^−1^ during which it exposes susceptible individuals at rate (1 − *o*_*S*_*c*_*S*_)(1 − *o*_*I*_*c*_*I*_)*β*, and the exposed individuals proceed to *I* with probability $\frac{\sigma }{\sigma +\tau }$.

It follows that the disease-free equilibrium (1, 0, 0, 0) with (*c*_*S*_, *c*_*T*_, *c*_*I*_) = (0, 0, 0) is stable when

$$\begin{array}{c} \beta (\frac{\sigma }{\sigma +\tau })(\frac{1}{\omega +\tau })<1,\text{and} \end{array}$$ (29)

$$\begin{array}{c} \kappa_{I}o_{I}\beta +\phi n_{F}-C_{W}<0 \end{array}$$ (30)

while the disease-free equilibrium (1, 0, 0, 0) with (*c*_*S*_, *c*_*T*_, *c*_*I*_) = (0, 0, 1) is stable when

$$\begin{array}{c} \left(1-o_{I}\right)\beta (\frac{\sigma }{\sigma +\tau })(\frac{1}{\omega +\tau })<1,\text{and} \end{array}$$ (31)

$$\begin{array}{c} \kappa_{I}o_{I}\beta +\phi n_{F}-C_{W}>0. \end{array}$$ (32)

### Endemic equilibria

We have to solve the following system of equations.

$$\begin{array}{c} 0=\rho T+\tau E-\left(1-o_{S}c_{S}\right)\left(1-o_{I}c_{I}\right)\beta SI \end{array}$$ (33)

$$\begin{array}{c} 0=\left(1-o_{S}c_{S}\right)\left(1-o_{I}c_{I}\right)\beta SI-\left(\sigma +\tau \right)E \end{array}$$ (34)

$$\begin{array}{c} 0=\sigma E+\left(1-o_{T}c_{T}\right)\left(1-o_{I}c_{I}\right)\Gamma \beta TI-\left(\omega +\tau \right)I \end{array}$$ (35)

$$\begin{array}{c} 0=\left(\omega +\tau \right)I-[\left(1-o_{T}c_{T}\right)\left(1-o_{I}c_{I}\right)\Gamma \beta I+\rho ]T \end{array}$$ (36)

$$\begin{array}{c} 0=\kappa^{\prime }c_{S}\left(1-c_{S}\right)(\kappa_{S}o_{S}\left(1-o_{I}c_{I}\right)\beta I-C_{W}) \end{array}$$ (37)

$$\begin{array}{c} 0=\kappa^{\prime }c_{T}\left(1-c_{T}\right)(\kappa_{T}o_{T}\Gamma \left(1-o_{I}c_{I}\right)\beta I-C_{W}) \end{array}$$ (38)

$$\begin{array}{c} 0=\kappa^{\prime }c_{I}\left(1-c_{I}\right)(\kappa_{I}o_{I}\beta \left(S+\Gamma T\right)+\phi n_{F}-C_{W}) \end{array}$$ (39)

It follows from (37)–(39) that any of the eight combinations of *c*_*S*_ ∈ {0, 1}, *c*_*T*_ ∈ {0, 1}, and *c*_*I*_ ∈ {0, 1} are potentially possible and there may also be other equilibria.

#### Case when *c*_*S*_, *c*_*T*_, *c*_*I*_ are known and all in {0, 1}

Adding (33) and (34) yields

$$\begin{array}{c} \rho T=\sigma E \end{array}$$ (40)

When *c*_*I*_ and *c*_*T*_ are known, it follows from (36) that

$$\begin{array}{c} T=\frac{(\omega +\tau )I}{\left(1-o_{T}c_{T}\right)\left(1-o_{I}c_{I}\right)\Gamma \beta I+\rho }. \end{array}$$ (41)

Thus

$$\begin{array}{c} E=\frac{\rho }{\sigma }\frac{(\omega +\tau )I}{\left(1-o_{T}c_{T}\right)\left(1-o_{I}c_{I}\right)\Gamma \beta I+\rho }. \end{array}$$ (42)

By (33),

$$\begin{array}{c} 0=\left(1-o_{S}c_{S}\right)\left(1-o_{I}c_{I}\right)\beta SI-\left(\sigma +\tau \right)\frac{\rho }{\sigma }\frac{(\omega +\tau )I}{\left(1-o_{T}c_{T}\right)\left(1-o_{I}c_{I}\right)\Gamma \beta I+\rho }. \end{array}$$ (43)

Thus, when *I* ≠ 0,

$$\begin{array}{cc} S & =\frac{\sigma +\tau }{\sigma }\frac{\rho (\omega +\tau )}{(\left(1-o_{T}c_{T}\right)\left(1-o_{I}c_{I}\right)\Gamma \beta I+\rho )\left(1-o_{S}c_{S}\right)\left(1-o_{I}c_{I}\right)\beta } \end{array}$$ (44)

$$\begin{array}{cc} & =\frac{\rho }{R}\frac{1}{\left(1-o_{T}c_{T}\right)\left(1-o_{I}c_{I}\right)\Gamma \beta I+\rho }. \end{array}$$ (45)

Thus,

$$\begin{array}{cc} 1 & =S+E+I+T \end{array}$$ (46)

$$\begin{array}{cc} & =\frac{\rho }{R}\frac{1}{\left(1-o_{T}c_{T}\right)\left(1-o_{I}c_{I}\right)\Gamma \beta I+\rho }+(\frac{\rho }{\sigma }+1)\frac{(\omega +\tau )I}{\left(1-o_{T}c_{T}\right)\left(1-o_{I}c_{I}\right)\Gamma \beta I+\rho }+I \end{array}$$ (47)

Consequently, *I* solves a quadratic equation

$$\begin{array}{c} 0=AI^{2}+BI-C. \end{array}$$ (48)

where

$$\begin{array}{c} A=\left(1-o_{T}c_{T}\right)\left(1-o_{I}c_{I}\right)\Gamma \beta \end{array}$$ (49)

$$\begin{array}{c} B=\frac{\rho (\omega +\tau )}{\sigma }+\omega +\tau +\rho -\left(1-o_{T}c_{T}\right)\left(1-o_{I}c_{I}\right)\Gamma \beta \end{array}$$ (50)

$$\begin{array}{c} C=\rho (1-\frac{1}{R}). \end{array}$$ (51)

The right-hand side of (48) is $-\rho (1-\frac{1}{R})$ for *I* = 0 and it is $\frac{\rho (\omega +\tau )}{\sigma }+\omega +\tau >0$ when *I* = 1. So there is a root *I* ∈ (0, 1) whenever R>1. Once *I* is calculated as the positive root of (48), we get the equilibrium values of the remaining compartments by

$$\begin{array}{c} S=\frac{\rho }{R}\frac{1}{\left(1-o_{T}c_{T}\right)\left(1-o_{I}c_{I}\right)\Gamma \beta I+\rho } \end{array}$$ (52)

$$\begin{array}{c} T=\frac{(\omega +\tau )I}{\left(1-o_{T}c_{T}\right)\left(1-o_{I}c_{I}\right)\Gamma \beta I+\rho } \end{array}$$ (53)

$$\begin{array}{c} E=\frac{\rho }{\sigma }T. \end{array}$$ (54)

### Cases when *c*_*S*_, *c*_*T*_, *c*_*I*_ are not all either 0 or 1

The Eqs (37)–(39) can also be satisfied without all *c*_*S*_, *c*_*T*_, *c*_*I*_ being in {0, 1}.

First, let us assume that *c*_*I*_ ∈ {0, 1}. If

$$\begin{array}{c} I=\frac{C_{W}}{\kappa_{S}o_{S}\left(1-o_{I}c_{I}\right)\beta }, \end{array}$$ (55)

then (37) holds. Unless there is an unlikely coincidence of parameter values, specifically unless *κ*_*S*_*o*_*S*_ = *κ*_*T*_*o*_*T*_Γ, (38) will hold only when *c*_*T*_ ∈ {0, 1}. We can then use (55) to calculate *T* by (53) and *E* by (55). Then *S* = 1 − (*E* + *I* + *T*) and we can use (33) to evaluate *c*_*S*_ as

$$\begin{array}{c} c_{S}=\frac{1}{o_{S}}(1-\frac{\rho T+\tau E}{(1-o_{I}c_{I})\beta SI}). \end{array}$$ (56)

We may do four different calculations, one for each combination *c*_*T*_, *c*_*I*_ ∈ {0, 1} and then select the appropriate equilibrium as described below in Section Selecting equilibria.

Similarly, when *c*_*I*_ ∈ {0, 1} and

$$\begin{array}{c} I=\frac{C_{W}}{\kappa_{T}o_{T}\left(1-o_{I}c_{I}\right)\beta \Gamma }, \end{array}$$ (57)

then (38) holds. Again, unless there is an unlikely coincidence of parameter values, specifically *κ*_*S*_*o*_*S*_ = *κ*_*T*_*o*_*T*_Γ, (37) will hold only when *c*_*S*_ ∈ {0, 1}. We can use (57) and (48) to calculate *c*_*T*_ by

$$\begin{array}{c} c_{T}=\frac{1}{o_{T}}(1-\frac{-\rho (1-\frac{1}{R})+[\frac{\rho (\omega +\tau )}{\sigma }+\omega +\tau +\rho ]I}{\left(1-o_{I}c_{I}\right)\Gamma \beta \left(I-I^{2}\right)}). \end{array}$$ (58)

Then we can use (52)–(54) to find *S*, *T*, and *E*. As above, we may need to do four different calculations, one for each combination *c*_*S*_, *c*_*I*_ ∈ {0, 1} and then select the appropriate equilibrium as described below in Section Selecting equilibria.

Finally, when *c*_*I*_ ≠ 0, we must have

$$\begin{array}{c} S+\Gamma T=\frac{C_{W}-\phi n_{F}}{\kappa_{I}o_{I}\beta }. \end{array}$$ (59)

Unless there is an unlikely coincidence in the values of the parameters, we can assume *c*_*S*_, *c*_*T*_ ∈ {0, 1}. Thus, we can use (48) to express *I* in terms of (1 − *o*_*I*_*c*_*I*_) and then (52) and (53) to express *S* and *T* in terms of (1 − *o*_*I*_*c*_*I*_). Substituting those into (59) yields an equation for *c*_*I*_ that can be solved.

### Selecting equilibria

The above procedure identifies many candidates for equilibria. Many of the candidates may have values outside of the biologically reasonable range between 0 and 1 and we need to disregard those. Yet, there is typically still a large number of potential equilibria that remains as there are always at least eight disease-free equilibria, one for every combination of *c*_*S*_, *c*_*T*_, *c*_*I*_ ∈ {0, 1}.

**Fig 2:**
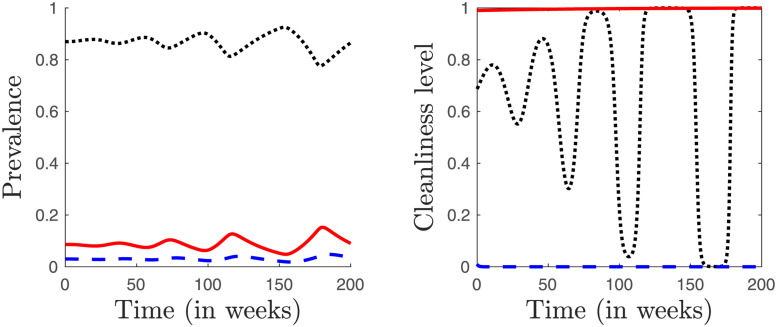
While rare (numerical experiments suggest it occurs in less than 0.1% cases) there is a possibility for multiple equilibria of the dynamics (7)–(10) and (17)–(19). For example, when the parameter values are as in Table 1 except *C*_*W*_ = 0.203, then there are three endemic equilibria $\left(S^{*},E^{*},I^{*},T^{*},c_{S}^{*},c_{T}^{*},c_{I}^{*}\right)$: (a) ≈ (0.364, 0.062, 0.44, 0.134, 1.0, 1.0, 0), (b) ≈ (0.563, 0.045, 0.294, 0.097, 1.0, 1.0, 0.39), and (c) ≈ (0.87, 0.014, 0.086, 0.03, 0.687, 0, 1.0). Numerical simulations suggest that the first two are locally stable while the last one is not as demonstrated by the figure above which shows dependence of the prevalence (left) and cleanliness level (right) on time. *S* and *c*_*S*_ shown by black dots, *I* and *c*_*I*_ by red solid line, *T* and *c*_*T*_ by blue dashed line. The initial condition was within 10^−2^ of each coordinate of the endemic equilibrium.

We did the following numerical experiment to determine the possibility of multiple stable equilibria. We used the Latin hyper-cube sampling that is typically used for sensitivity analysis [45–48]. It is a stratified sampling without replacement; the parameters were drawn from their ranges as specified in Table 1; the ranges were divided into intervals of equal lengths and the sampling was independent for each parameter. This method provides an unbiased estimate of the average model output while it requires fewer samples than simple random sampling to achieve the same accuracy [49]. For each of the 10000 sets of the sampled parameter values, we calculated all solutions of the Eqs (33)–(39). Then we discarded solutions outside of the [0, 1] range. Finally, we looked at the signs of the right-hand sides of (17)–(19). If the right hand side of (17) was positive and $c_{S}^{*}$ was less than 1, we disregarded this solution as unstable because susceptible individuals have incentive to use water more; similarly, we disregarded solution with $c_{S}^{*}>0$ when the right hand side of (17) was negative. A similar procedure was applied to *c*_*T*_ and *c*_*I*_ as well. We tracked the number of solutions that were left after the discarding procedure and have seen only seven combinations of parameters that resulted in more than one equilibrium. One such combination is shown in Fig 2, the remaining parameter sets produced only one set of equilibria.

It turned out that the parameter combinations yielding multiple equilibria all corresponded to the narrow bands of transitions from *c*_*I*_ = 1 to *c*_*I*_ = 0 as *C*_*W*_ grew; these transitions are further discussed in Section Results. In all cases, there were three endemic equilibria, one with $c_{I}^{*}=1$, one with $c_{I}^{*}=0$, and one with $c_{I}^{*}\in \left(0,1\right)$. While interesting from mathematical perspective, the multiple equilibria occur for such a narrow range of parameters (by (59), one needs |*C*_*W*_ − *ϕn*_*F*_| < *κ*_*I*_*o*_*I*_*β*) that they can be effectively disregarded for practical purposes.

## Calibration

For the disease progression, we will use values derived in [25, Table 2]. The incubation period, *σ*^−1^ is 17.4 days, i.e., about 2.5 weeks (95% CI: 11–28 days). The infection, i.e., PCR positivity, the stages *E* and *I* together) lasts 17.2 weeks (95% CI: 13.7 to 23 weeks). This means the duration of the infectious period, *ω*^−1^, is given about 17.2 − *σ*^−1^ ≈ 15 weeks with the range of about 10 to 21.5 weeks. The post infection inflammation period, *ρ*^−1^, is about 5.4 weeks (95% CI: 3.6 to 8.0 weeks). We note that the active disease, i.e., TF positivity, *I* and *T* together is about 21 weeks [25] which reasonably agrees with our estimate $\omega^{-1}+\rho^{-1}$.

To estimate *β*_*H*_, we used [24] who, in their supplement file, presented estimates of *β*_*H*_ for their model 2 based on susceptibility of the population. In this paper, we assume Γ = 0.5 which, by [24], yields *β*_*H*_ = 0.061 with the range [0.05, 0.083] per day, i.e., *β* ≈ 0.43 with the range [0.35, 0.58] per week.

We assume that the number of flies per person is *n*_*F*_ = 5. Similarly, to achieve 50% transmission, we assume *n*_*F*,1/2_ = 2. For simplicity, we assume that flies can transmit trachoma as much as the people and we set $\beta_{F}^{\text{max}}=0.43$. But we will also consider the scenario when the flies actually do not transmit trachoma at all, i.e. when $\beta_{F}^{\text{max}}=0$.

[37] conducted a systematic survey of literature on the effect of hygiene on the prevention of trachoma. In particular, their Figure 14 shows the association of washing face more than once per day (versus less than once per day) with TF/TI. The mean odds ratio is 0.76 with 95% CI [0.57, 0.99]. Washing face twice or more per day did not show too significant improvement. Similar odds were found for bathing or using soap. The towel use lowered the odds ratio slightly to 0.65. In light with these data, we will thus assume *o*_*S*_ = *o*_*T*_ = *o*_*I*_ = 0.76 with range [0.57, 0.99].

As in [50] we are interested in what happens after the application of MDA, i.e. when *τ* = 0. If we wanted to study the situation during the MDA rounds, we would use *τ* = 0.0155 because MDA are done annually, i.e. at rate 1/52 per week, each MDA round had a population coverage level 85% and antibiotic efficacy of 95% [51]; yielding *τ* ≈ 1/52 × 0.85 × 0.95.

In the region where trachoma is prevalent, water can be a very scarce resource and the access to water is often as far as 30 minutes away or further [52]. Furthermore, Figure 2 from [52] shows that the per capita per day consumption of water remains largely independent of the time one needs to collect the water as long as the time is roughly 5–30 minutes. We can thus assume that the cost of water is proportional to the time needed to acquire it. Just for scale, we set *C*_*W*_ = 1 but we will keep in mind that the cost is variable.

For the numerical simulations, we also have to set the scaling constants *κ*_*S*_, *κ*_*T*_, *κ*_*I*_ and *κ*′. We set *κ*_*S*_ = *κ*_*T*_ = 20 to convert the risk of infection into the cost. Also, we set *κ*_*I*_ = 0.01 to include some (but not large) inclusive fitness costs for the infectious individuals to infect others. And we set *κ*′ = 10 to specify that the behavioral dynamics is happening roughly on a ten times faster scale than the trachoma transmission.

Finally, *ϕ* is a factor that converts the number of flies per person, *n*_*F*_, into the discomfort cost *ϕn*_*F*_ (as the flies are attracted to the ocular discharge of the infectious cases). We set *ϕ* = 0.1 and vary the factor between 0 and 0.15.

## Results

**Fig 3:**
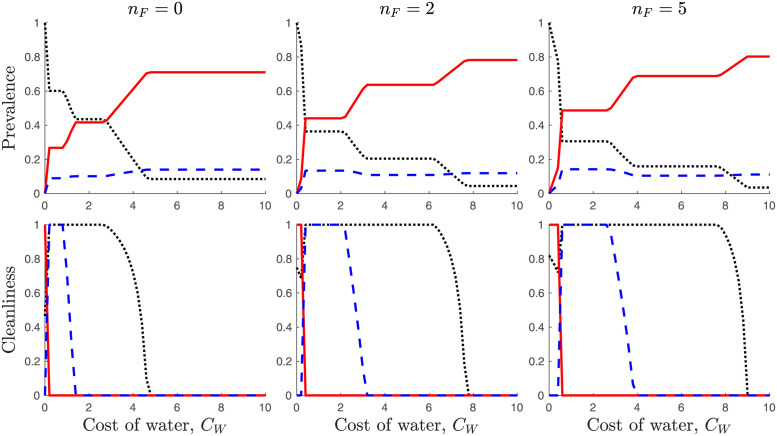
Top row: Dependence of prevalence of *S* (black dots), *I* (red solid line), and *T* (blue dashed line) on the cost of water *C*_*W*_. Bottom row: Dependence of cleanliness levels *c*_*S*_ (black dots), *c*_*I*_ (red solid line), and *c*_*T*_ (blue dashed line) on the cost of water *C*_*W*_. Left column is for *n*_*F*_ = 0, the middle column is for *n*_*F*_ = 2 and the right column is for *n*_*F*_ = 5. Unless varied or specified, the parameters are as in Table 1.

**Fig 4:**
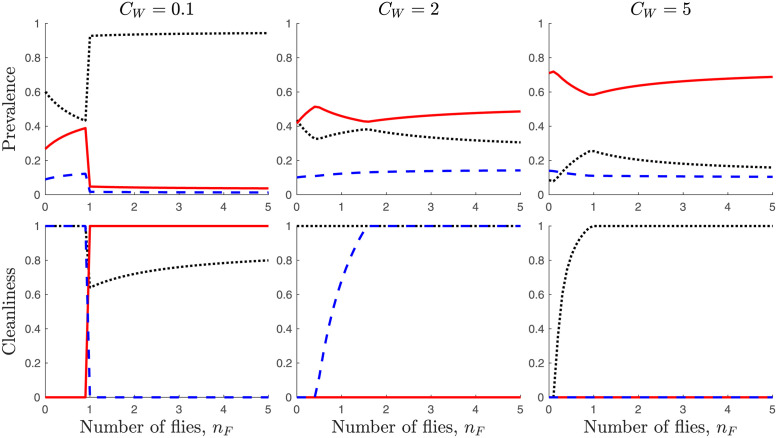
Top row: Dependence of prevalence of *S* (black dots), *I* (red solid line), and *T* (blue dashed line) on the number of flies per person, *n*_*F*_. Bottom row: Dependence of cleanliness levels *c*_*S*_ (black dots), *c*_*I*_ (red solid line), and *c*_*T*_ (blue dashed line) on the number of flies per person, *n*_*F*_. Left column is for *n*_*F*_ = 0 while the right column is for *n*_*F*_ = 5. Unless varied or specified, the parameters are as in Table 1.

We investigate how the equilibrium values depend on the cost of water (Fig 3) and the number of flies (Fig 4).

**Fig 5:**
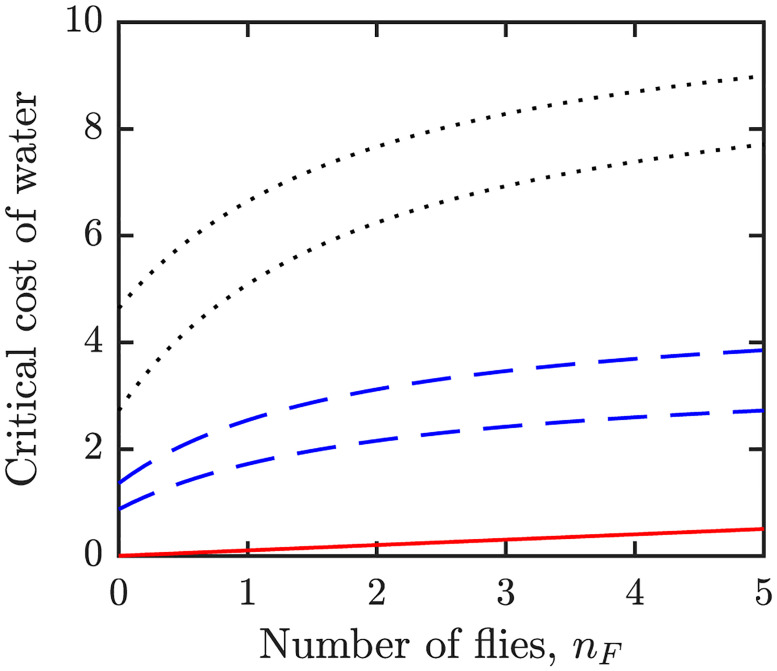
The dependence of the critical transitions $C_{W}^{I}$ (red solid line), $C_{W}^{T}$ (blue dashed lines), and $C_{W}^{S}$ (black dotted lines) on *n*_*F*_. *c*_*I*_ decreases from 1 and *c*_*T*_ increases from 0 at $C_{W}^{I}$. *c*_*T*_ decreases from 1 to 0 as *C*_*W*_ increases from the bottom blue dashed line top blue dashed line. *c*_*S*_ decreases from 1 to 0 as *C*_*W*_ increases from the lower black dotted line to the top black dotted line. During each of these transitions, I* is increasing in *C*_*W*_.

When the cost of water is low, all infectious individuals prefer to use it as a defense against the flies. This results in relatively small risks of infection for individuals in *S* and *T*. Consequently, only some individuals in *S* and none in *T* need to use water for cleanliness. However, as the cost of water increases above a threshold $C_{W}^{I}$, which is by (59), for small *κ*_*I*_ approximately given by

$$\begin{array}{c} C_{W}^{I}=\kappa_{I}o_{I}\beta +\phi n_{F}, \end{array}$$ (60)

then it is no longer beneficial for individuals in *I* to practice cleanliness. This results in a sharp increase in the prevalence of infectious cases. At the same time, all individuals in *S* and *T* now have a strong incentive to practice cleanliness. As the cost of water grows even further, using it for cleanliness starts to outweigh the risks of reinfection for *T* cases who eventually stop practicing cleanliness. By (57), this happens at

$$\begin{array}{c} C_{W}^{T}=\kappa_{T}o_{T}\beta \Gamma I \end{array}$$ (61)

where, for *c*_*S*_ = 1, *c*_*I*_ = 0, and *c*_*T*_ varying from 1 to 0, *I* solves (48). This results in another increase of infectious cases. Interestingly, this also results in a slight decline of the proportion of *T* cases. Finally, as the cost of water increases even further, it outweighs the risk of infection for the susceptible individuals. Thus, even susceptible individuals eventually stop practicing cleanliness altogether, which results in the final increase of infectious cases. By (57), this last transition happens at

$$\begin{array}{c} C_{W}^{S}=\kappa_{S}o_{T}\beta I \end{array}$$ (62)

where *I* solves (48) for *c*_*T*_ = 0, *c*_*I*_ = 0 and *c*_*S*_ varying from 1 to 0. These results are illustrated in Fig 3. The dependence of the critical transitions $C_{W}^{I},C_{W}^{T}$, and $C_{W}^{S}$ on *n*_*F*_ is shown in Fig 5.

**Fig 6:**
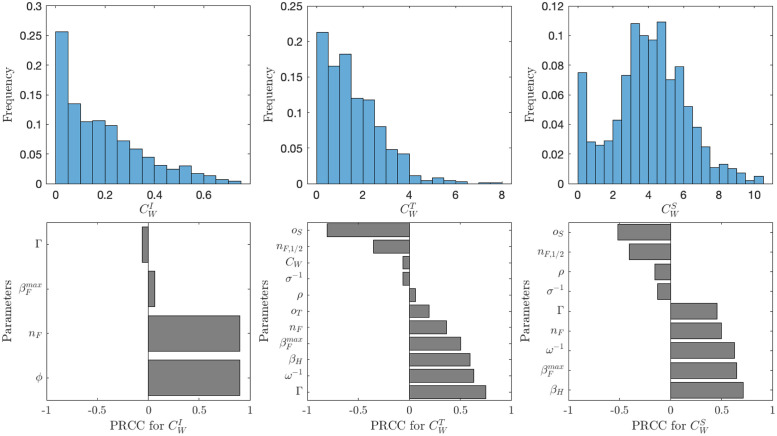
The distribution (top row) and sensitivity analysis (bottom row) of the threshold values $C_{W}^{I}$ (left), $C_{W}^{T}$ (middle) and $C_{W}^{S}$ (right). We used the Latin hyper-cube sampling with partial rank correlation coefficient (LHS-PRCC) scheme [45, 46] described in detail in [47] with the MATLAB and R implementation in [48]. In the top row, the “peaks” at 0 correspond to disease-free equilibria. For the endemic equilibria, $C_{W}^{T}$ peaks around 1.75 and $C_{W}^{S}$ peaks around 4.5. In the bottom row, only parameters with significant PRCC coefficients (in absolute value greater than 0.05 are shown). The parameter values ranges are as specified in Table 1.

The uncertainty and sensitivity analysis of the threshold values $C_{W}^{I},C_{W}^{T},C_{W}^{S}$ is shown in Fig 6. By (60), $C_{W}^{I}$ is increasing in *ϕ* and *n*_*F*_ and there is minimal (typically not significant enough and thus not shown in Fig 6 on *o*_*I*_ and *β*, i.e., on $\beta_{H},\beta_{F}^{\text{max}}$). The thresholds $C_{W}^{T}$ and $C_{W}^{S}$ generally depend on the parameters in the similar way: they both increase in *n*_*F*_, *β*_*H*_, $\beta_{F}^{\text{max}}$, *ω*^−1^ and Γ and decrease in *o*_*S*_ and *n*_*F*,1/2_.

**Fig 7:**
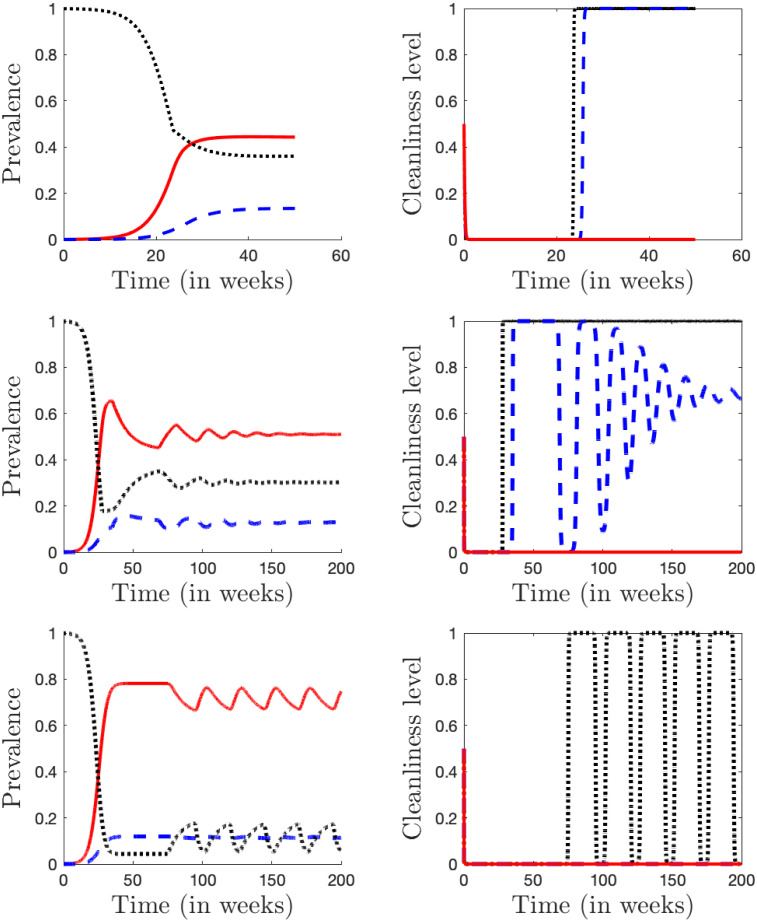
Dependence of the prevalence (left) and cleanliness level (right) on time. The curves represent solutions of the ODEs (7)–(10) and (17)–(19). *S* and *c*_*S*_ shown by black dots, *I* and *c*_*I*_ by red solid line, *T* and *c*_*T*_ by blue dashed line. The initial condition was near disease-free equilibrium with *S* = 0.999, *E* = 0.001, *I* = 0, *T* = 0 and *c*_*S*_ = *c*_*T*_ = *c*_*I*_ = 0.5. Unless otherwise noted, the parameters are as in Table 1. **Top row**: *C*_*W*_ = 1; The values converge to the endemic equilibrium $\left(S^{*},E^{*},I^{*},T^{*},c_{S}^{*},c_{I}^{*},c_{T}^{*}\right)\approx \left(0.364,0.062,0.44,0.133,1,0,1\right)$ with an abrupt change around weeks 23–26 when the number of infectious cases is high enough for the susceptible and then even for *T* to use water for facial cleanliness. **Middle row**: *C*_*W*_ = 2.5; the values converge to an endemic equilibrium ≈(0.302, 0.06, 0.51, 0.129, 1.0, 0.683, 0). **Bottom row**: *C*_*W*_ = 7; the values do not converge to the endemic equilibrium ≈(0.118, 0.053, 0.714, 0.115, 0.804, 0, 0).

There are several qualitative differences between the transitional and non-transitional regions. The first transition around $C_{W}^{I}$ yields multiple equilibria; although as discussed earlier, the transition is too narrow to have any practical significance. The transition around $C_{W}^{S}$ yields an endemic equilibrium with $c_{S}^{*}\in \left(0,1\right)$ that is not locally asymptotically stable. The transition around $C_{W}^{T}$ yields an equilibrium with $c_{T}^{*}\in \left(0,1\right)$ that appears stable, but the convergence to the equilibrium is slower than in the case of parameters completely outside of the transition regions and when all $c_{S}^{*},c_{T}^{*},c_{I}^{*}\in \left\{0,1\right\}$, see Figs 2 and 7.

The dependence of the equilibrium values on the number of flies per person, *n*_*F*_, is also interesting as shown in Fig 4. When there are not many flies, the infectious individuals prefer not to use water for cleanliness and, as a result, the prevalence of *I* increases with *n*_*F*_. However, as the number of flies crosses a threshold $n_{F}^{I}$, which, for small *κ*_*I*_, is given approximately by

$$\begin{array}{c} n_{F}^{I}=\frac{1}{\phi }\left(C_{W}-\kappa_{I}o_{I}\beta \right), \end{array}$$ (63)

the flies constitute an annoyance to infectious individuals who start using water for cleanliness. This shift to cleanliness causes a sharp decline of infections. This lowers the risk of reinfections for *T* who stop practicing cleanliness; individuals in *S* also reduce their cleanliness levels. This is illustrated in the left column of Fig 4 when the cost of water is not too high. When the cost of water is higher, as in the middle column of Fig 4, the infectious individuals do not have incentives to practice cleanliness (unless the number of flies reaches beyond the threshold $n_{F}^{IH}$ given in (63)). However, as the number of flies increases, the risk of reinfection in *T* increases which causes those individuals to start practicing cleanliness. This yields a decline in infectious cases. Similarly, for even higher cost of water (as shown in the right column of Fig 4), the increase of flies causes the rise of the risk of infection in *S*. This results in susceptible individuals increasing their cleanliness efforts. Consequently, the number of infectious individuals decreases with the number of flies. However, once all susceptible individuals practice cleanliness, the number of infectious cases rises with the number of flies.

## Conclusions and discussion

In this paper, we incorporated human behavior into an epidemiological model of trachoma. We allow individuals practice facial cleanliness based on their epidemiological status and their perceived benefits and costs. We were specifically interested in studying how the optimal behavior depends on the cost of water (assumed to be correlated with the time it takes to collect water) and the number of flies per person. We found that the number of infectious individuals generally increases with the difficulty to access a water source. However, this increase happens only during three transition periods and the prevalence stays constant otherwise.

The first transition happens at relatively low costs of water; during this transition the infectious individuals stop using water for their facial cleanliness which results in sharp increase of infectious and trachoma cases. While our model is qualitative rather than quantitative in nature, we believe that this transition corresponds to the sharp decline in water consumption observed in [52] when the time to collect water increases from 0 to less than 5 minutes.

The second transition occurs for medium water costs and coincides with trachoma cases stopping to use water as a prevention against reinfection. This results in another increase of infectious cases. Ironically, the prevalence of trachoma cases declines (when there are enough flies), but this is due to the fact that those cases are getting reinfected rather than the disease being eliminated. When there are not many flies present in the population, the prevalence of trachoma disease increases together with the cost of water.

Finally, the third transition occurs for even larger water costs when the cost is prohibitive even for the susceptible individuals to use it as a prevention against contracting trachoma. This transition results in further increase of infectious cases, yet does not significantly affect the prevalence of trachoma disease.

Overall, the above results of our qualitative model agree with [52] who notes that the effect of distance to water is contradictory. Furthermore, we see, similarly as in [53] that there is a positive correlation between a distance to the water source and the prevalence of active trachoma.

From the public health policy perspective, our model predicts that while the access to water is important, improving the access to water does not automatically lower the prevalence of trachoma. In fact, and this is particularly demonstrated in Fig 3, the cost of water has most of the time no effect on the trachoma prevalence, it is only during the three transition periods that prevalence changes. Consequently, for the improved access to have a measurable effect on trachoma prevalence, it should be an improvement that is significant enough that it crosses the transition thresholds.

There are several ways our model can be improved further and extended in a way that it could be used to make quantitative rather than just qualitative predictions. First, one should include an appropriate age structure as done, for example, in [24]. We left our model simpler in order to keep the necessary number of parameters as low as possible. Second, one may need a better understanding of the behavioral dynamics. That includes appropriately estimating the speed at which the behavioral changes happen as well as better understanding how individuals evaluate the risks of trachoma versus the difficulty of water access. Third, we assumed a homogeneous population and this assumption may not be realistic for several reasons as individuals may vary with their access to water and their knowledge about the disease which would potentially alter their behavior. Consequently, a simulation and individual based models may be more appropriate tools for more detailed investigations.

## Supporting information

S1 File (M)
